# Thalidomide for CGD-related inflammatory bowel disease: A randomized, double-blind trial

**DOI:** 10.70962/jhi.20250256

**Published:** 2026-06-23

**Authors:** Toshinao Kawai, Mayumi Sako, Katsuhiro Arai, Takashi Ishikawa, Tomoko Toma, Taizo Wada, Yutaro Yada, Masataka Ishimura, Takehiko Doi, Satoshi Okada, Hiroshi Moritake, Kazushi Izawa, Masahiro Ueki, Eisuke Inoue, Hidefumi Nakamura, Masafumi Onodera

**Affiliations:** 1 Division of Immunology, National Center for Child Health and Development, Tokyo, Japan; 2 Department of Clinical Research Promotion, Clinical Research Center, National Center for Child Health and Development, Tokyo, Japan; 3 Division of Gastroenterology, National Center for Child Health and Development, Tokyo, Japan; 4 Department of Pediatrics, School of Medicine, Institute of Medical, Pharmaceutical and Health Sciences, Kanazawa University, Kanazawa, Japan; 5 Department of Pediatrics, Graduate School of Medical Sciences, Kyushu University, Fukuoka, Japan; 6 Department of Pediatrics, Hiroshima University Graduate School of Biomedical and Health Sciences, Hiroshima, Japan; 7 Division of Pediatrics, Faculty of Medicine, University of Miyazaki, Miyazaki, Japan; 8 Department of Pediatrics, Kyoto University Graduate School of Medicine, Kyoto, Japan; 9 Department of Pediatrics, Graduate School of Medicine, Hokkaido University, Sapporo, Japan; 10 Showa Medical University Research Administration Center, Showa Medical University, Tokyo, Japan; 11 Department of Research and Development Supervision, National Center for Child Health and Development, Tokyo, Japan

## Abstract

Chronic granulomatous disease–related inflammatory bowel disease (CGD-IBD) requires effective and safe therapies, as conventional immunosuppressants increase infection risk. Thalidomide, with anti-inflammatory and immunomodulatory effects, may represent a therapeutic option. This study was conducted to explore the preliminary efficacy and safety of thalidomide for CGD-IBD in a multicenter, randomized, double-blind, placebo-controlled, parallel-group phase II trial. Patients were randomized to thalidomide or placebo for a 12-wk blinded phase, followed by a 12-wk extension thalidomide phase. The primary endpoint was achievement of remission or a ≥20-point reduction in the Pediatric Ulcerative Colitis Activity Index. Eight male patients were randomized and analyzed. The primary endpoint was achieved by 1/3 of thalidomide patients versus 0/5 in the placebo group during the blinded phase, and by 5/8 in the extension phase. Secondary endpoints and exploratory endoscopy showed improvements. CGD-related infection rates were comparable before and after treatment. Thalidomide met the prespecified efficacy criterion with an acceptable safety profile in CGD-IBD, suggesting a promising therapeutic option that warrants further clinical studies.

## Teaser

In this first randomized, placebo-controlled trial, thalidomide demonstrated potential efficacy and acceptable safety in patients aged 1 year or older with chronic granulomatous disease–related inflammatory bowel disease, suggesting potential as a therapeutic option without increasing the risk of infection.

## Introduction

Chronic granulomatous disease (CGD) is an extremely rare inborn error of immunity caused by defective nicotinamide adenine dinucleotide phosphate oxidase complex in phagocytes, leading to impaired production of reactive oxygen species. Prevalence of CGD is about 1 in 225,000 live births in the United States and similar in Japan (1, 2). Besides recurrent bacterial and fungal infections (3, 4), CGD is complicated by hyperinflammation, resulting in granuloma formation, macrophage activation syndrome, and CGD-related inflammatory bowel disease (CGD-IBD). Nearly half of patients develop CGD-IBD during childhood or adulthood (5). Although its mechanisms differ from ulcerative colitis and Crohn’s disease, CGD-IBD presents with diarrhea, hematochezia, abdominal pain, and fever, similar to ulcerative colitis and Crohn’s disease (6, 7, 8, 9).

As in typical IBD, anti-inflammatory treatment is required for CGD-IBD (10). Corticosteroids, immunosuppressants (azathioprine, methotrexate, cyclosporine A), and biologics targeting tumor necrosis factor α (TNF-α) or interleukin-1 (IL-1) are used (11, 12, 13, 14). Although these agents improve symptoms, they also increase susceptibility to infections and pose a risk of fatal outcomes, which is unacceptable in CGD (13, 14). Recently, several reports have described clinical improvement in patients with CGD-IBD treated with non–anti-TNF-α biologics, including ustekinumab, although careful monitoring for infectious complications remains essential (15, 16). Thus, new therapeutic options that effectively control CGD-IBD symptoms without raising infection risk are urgently needed.

Thalidomide has unique, pleiotropic medical effects, including anti-angiogenesis, anti-TNF-α, and immunomodulation. Unlike anti-TNF-α monoclonal antibodies such as infliximab and adalimumab, which neutralize circulating TNF-α, thalidomide suppresses TNF-α production by monocytes/macrophages and is considered less likely to impair host immune responses against bacterial infections (17, 18). Furthermore, clinical trials in patients with inflammatory diseases have not reported an increased frequency of bacterial or fungal infections associated with thalidomide treatment (19, 20, 21). Thalidomide has been approved in the United States, Europe, and Japan as a capsule formulation for multiple myeloma and erythema nodosum leprosum. Although its pharmacological mechanisms are not fully elucidated, it is known to exert anti-angiogenic, TNF-α–blocking, and immunomodulatory effects, with no known ethnic differences in efficacy or safety (22, 23, 24). Randomized controlled trials showed the efficacy of thalidomide in pediatric ulcerative colitis and Crohn’s disease (20, 21). There have been case reports describing the use of thalidomide in which CGD-IBD symptoms improved when thalidomide was combined with corticosteroids or immunosuppressants (9, 17, 25, 26). Given the role of dysregulated TNF-α in CGD-IBD (14), thalidomide may control inflammation without increasing infection risk. Previously, the efficacy of therapeutic agents for CGD-IBD has primarily been evaluated based on abdominal symptoms, such as abdominal pain, hematochezia, and stool consistency; however, no reports have systematically quantified the natural course of these clinical parameters using validated scoring systems and analyzed them in detail. We therefore conducted a randomized, double-blind trial in patients aged ≥1 year with CGD-IBD to explore the preliminary efficacy and safety of thalidomide with placebo.

## Results

### Patients

Nine patients underwent screening, but one withdrew consent due to receiving immunosuppressive treatment that met the exclusion criteria. Consequently, eight patients were enrolled between December 6, 2017, and June 11, 2022, and follow-up of the last patient concluded on February 20, 2023. Three patients were 1 year old; one patient each was 6, 8, and 25 years old; and two patients were 28 years old (Table 1). All patients were Asian males of Japanese ethnicity and had a history of concomitant trimethoprim/sulfamethoxazole and antifungal use. For CGD-IBD severity, the Pediatric Ulcerative Colitis Activity Index (PUCAI) classified seven patients as moderate and one as mild at screening, and five as moderate and three as mild at baseline; by the Physician Global Assessment (PGA) at baseline, four patients were moderate and four were mild.

**Table 1.** Patient demographics and baseline characteristics

Parameter	Thalidomide (*n* = 3)			Placebo (*n* = 5)				
	T1	T2	T3	P1	P2	P3	P4	P5
Age, years	6	25	1	1	28	28	8	1
Sex	Male	Male	Male	Male	Male	Male	Male	Male
Genotype	*CYBB*	*CYBB*	*CYBB*	*CYBB*	*CYBB*	*CYBB*	*NCF2*	*CYBB*
Weight, kg	14.6	67.1	9.6	10.7	46.5	51.2	23.7	9.3
PUCAI at screening	45	40	35	40	35	35	30	40
PUCAI at 0 wk	15	45	25	50	35	40	35	25
PGA at screening	Moderate	Moderate	Moderate	Moderate	Moderate	Moderate	Mild	Mild
PGA at 0 wk	Mild	Moderate	Mild	Moderate	Moderate	Moderate	Mild	Mild
Infection at initiation of trial	–	–	–	–	–	–	–	Thymic abscess
Concomitant drugs at 0 wk	TMP-SMX	Meropenem	Cefditoren pivoxil	TMP-SMX	TMP-SMX	TMP-SMX	TMP-SMX	Cefmetazole
	Itraconazole	Itraconazole	Itraconazole	Fluconazole	Itraconazole	Itraconazole	Itraconazole	Micafungin

PUCAI, Pediatric Ulcerative Colitis Activity Index; PGA, Physician Global Assessment; TMP-SMX, trimethoprim/sulfamethoxazole.

Three patients (T1–3) were randomized to the thalidomide and five (P1–5) to the placebo; all received the assigned treatment in the blinded phase, followed by thalidomide in the extension phase with follow-up, ensuring 100% compliance for both phases. All eight patients were included in the efficacy and safety analysis. No corticosteroids or immunosuppressants were used during the trial. Dose escalation was performed according to the protocol; the starting dose, maximum and minimum doses, and the dose modification for the study treatment are shown in Table 2.

**Table 2.** Dose of the investigational drug and adverse events of bacterial and fungal infections by study phases

Phase	Parameter	Thalidomide (*n* = 3)			Placebo (*n* = 5)				
		T1	T2	T3	P1	P2	P3	P4	P5
Blinded phase	Infection	Aspergillosis CRP increase	None	Otitis media	None	None	None	None	Thymic abscess
				Pustular eczema					
				Lymphadenitis					
	Investigational drug dose at initiation, mg	30	130	20	20	90	100	50	20
	(min, max)	(30, 45)	(95, 130)	(20, 30)	(20, 30)	(90, 130)	(100, 150)	(50, 75)	(20, 30)
	Dose modification	Increased	Decreased	Increased	Increased	Increased	Increased	Increased	Increased
	Reason for modification	Insufficient efficacy	Drowsiness	Insufficient efficacy	Insufficient efficacy	Insufficient efficacy	Insufficient efficacy	Insufficient efficacy	Insufficient efficacy
Extension phase	Infection	None	Perirectal abscess	None	Balanoposthitis	Lymphadenitis	Infectious enteritis	–	Thymic abscess
						Esophageal candidiasis			
						Gastritis	Perirectal abscess		
	Thalidomide dose at initiation, mg	45	130	30	20	90	100	50	20
	(min, max)	–	–	–	(20, 30)	–	(100, 150)	–	(20, 30)
	Dose modification	–	–	–	Increased	–	Increased	–	Increased
	Reason for modification	–	–	–	Insufficient efficacy	–	Insufficient efficacy	–	Insufficient efficacy

The CGD-related infections included bacterial and fungal infections, but not viral infections. The table shows the CGD-related infections that developed or worsened during each phase. CRP, C-reactive protein.

**Figure S1.**
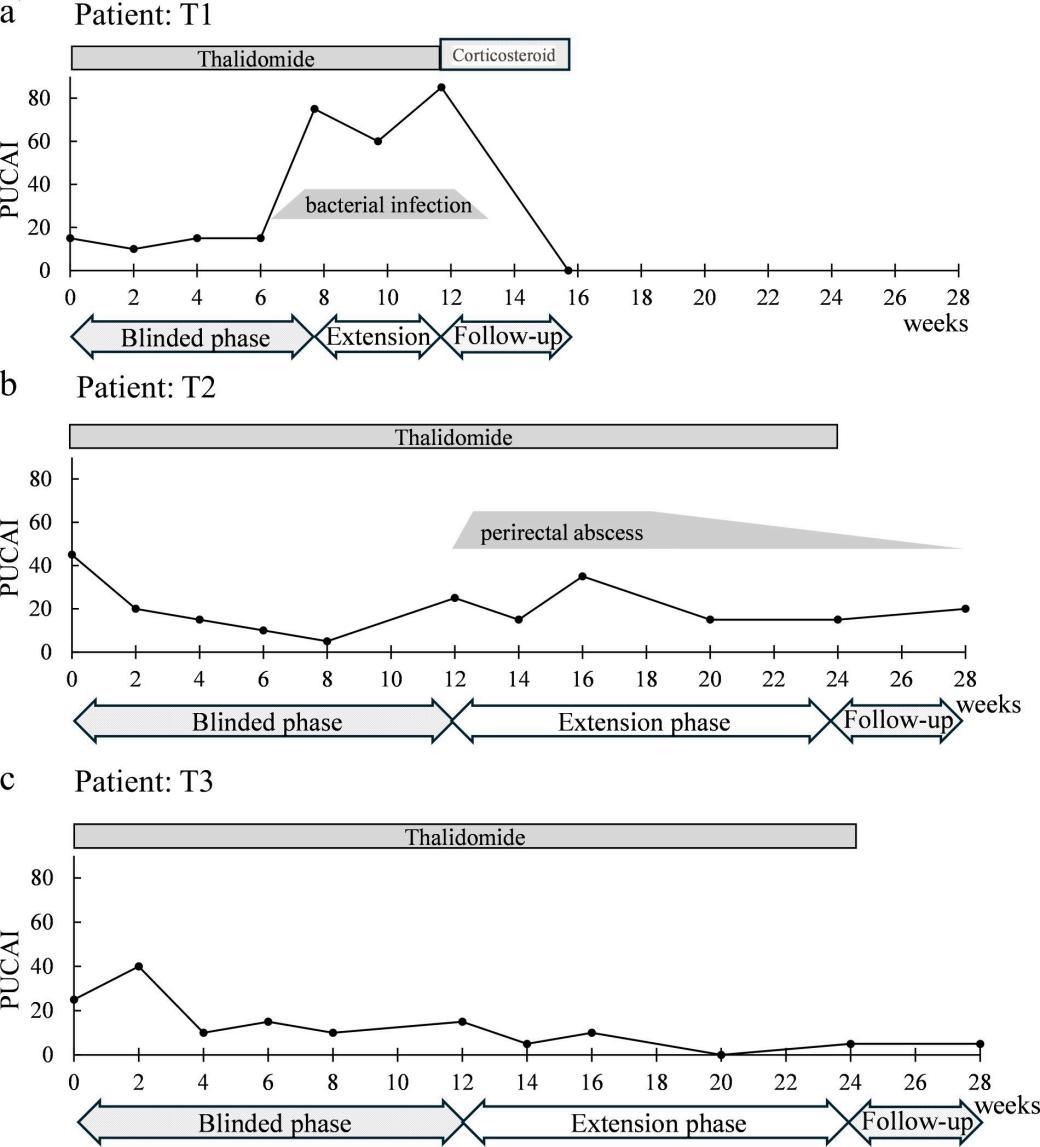
**Clinical course and PUCAI of patients in the thalidomide group. (a)** PUCAI increased with high C-reactive protein, bacterial infection suspected, at wk 6–8, leading to the blinded phase discontinuation in patient T1. With no improvement in the extension phase, the patient withdrew from the trial and was treated with corticosteroids. **(b)** PUCAI decreased by 20 points below baseline and met the primary endpoint in patient T2. **(c)** PUCAI decreased by 10 points below baseline, which did not meet the primary endpoint; however, clinical remission (PUCAI <10) was achieved during the extension phase in patient T3.

**Figure S2.**
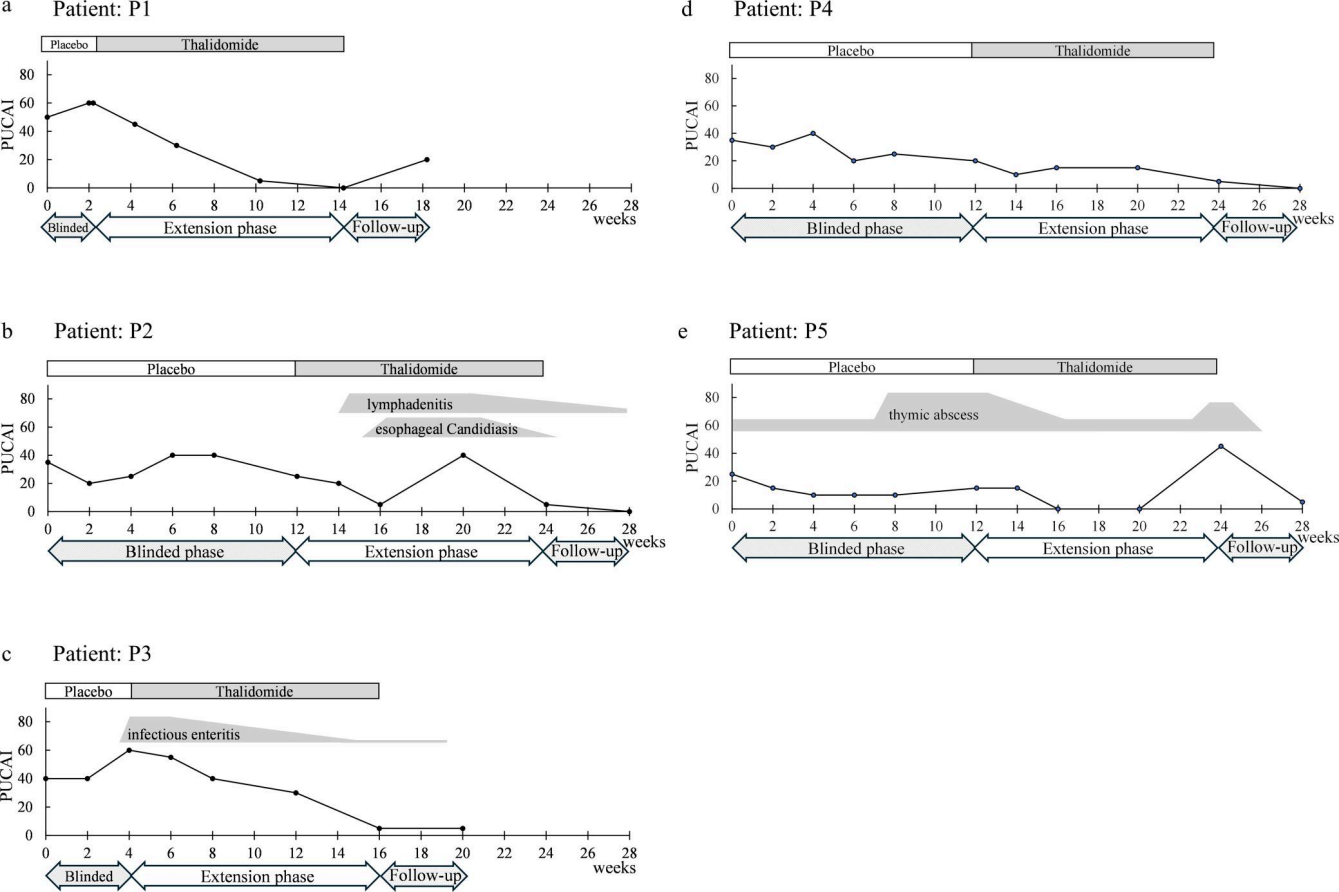
**Clinical course and PUCAI of patients in the placebo group. (a)** Following discontinuation of the blinded phase at wk 2, PUCAI decreased with thalidomide in the extension phase in patient P1. **(b)** During the infection, although PUCAI temporarily increased at wk 20, patient P2 achieved remission at the end of the extension phase. **(c)** Following discontinuation of the blinded phase at wk 4, PUCAI decreased with thalidomide in the extension phase in patient P3. **(d)** Remission was achieved at the end of the extension phase in patient P4. **(e)** Following remission at wk 16 and 20, PUCAI temporarily increased at wk 24 in patient P5. Subsequently, with improvement of the thymic abscess, PUCAI decreased to below 10 at the end of the follow-up period without further treatment for CGD-IBD.

**Figure 1.**
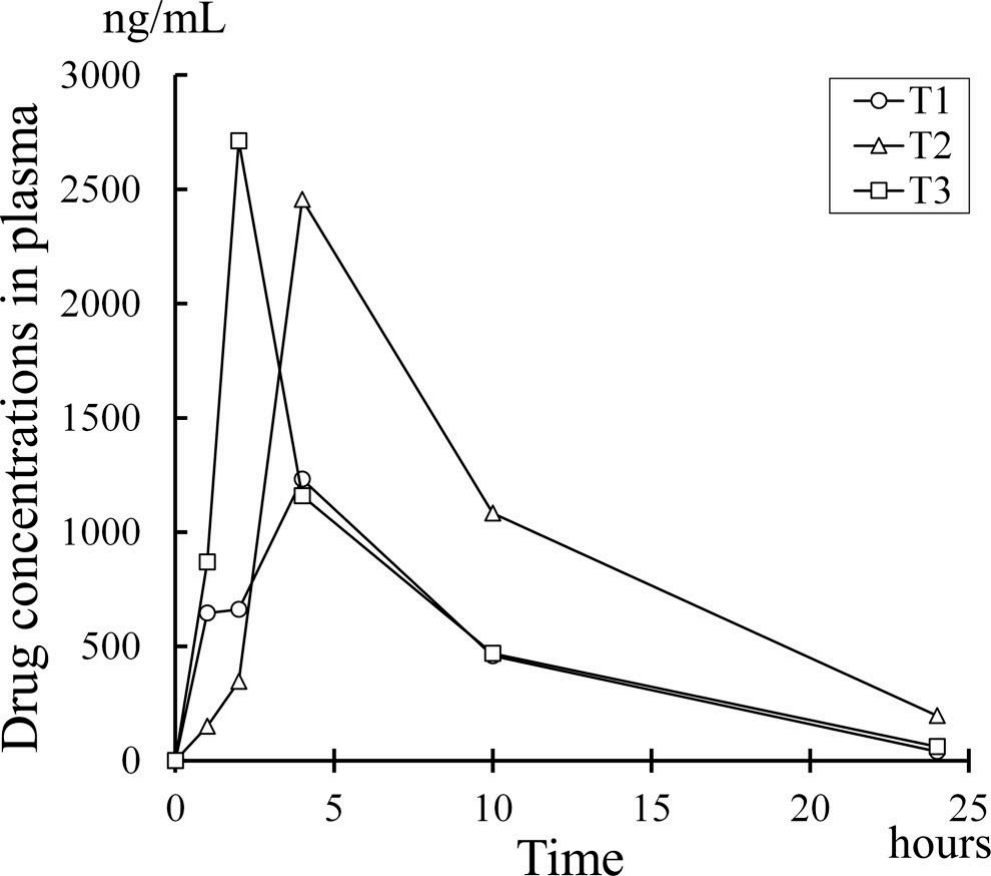
**Plasma drug concentrations in the thalidomide group.** Plasma drug concentrations following the initial administration of thalidomide were measured in two pediatric patients (T1 and T3) and one adult patient (T2) in the thalidomide group.

In the blinded phase, one thalidomide-treated patient (T1) developed a bacterial infection at wk 6, and by wk 8, his PUCAI had risen to 75, prompting transfer to the extension phase; however, as the infection persisted and his PUCAI increased to 85, treatment was discontinued at wk 12 (Fig. S1 a). PUCAI also rose to 60 in two placebo-treated patients (P1 and P3, Fig. S2, a and c), leading to discontinuation of the blinded phase and transfer to the extension phase. The remaining five patients completed 12 wk of the blinded phase and entered the extension phase (Table S1). Pharmacokinetic parameters in the thalidomide group (T1–3) and drug concentrations in each patient are presented in Table 3 and Fig. 1.

**Table 3.** Pharmacodynamics of thalidomide in the thalidomide group

Pharmacokinetic parameters	T1	T2	T3
Age, years	6	25	1
AUC_t_ (hr·ng/ml)	11,238.10	22,824.50	14,493.20
AUC_∞_(hr·ng/ml)	11,470.90	24,431.50	14,920.90
C_max_ (ng/ml)	1,232.20	2,454.70	2,711.90
t_max_ (h)	4.00	3.87	1.88
t_1/2_ (h)	4.06	5.65	4.87

AUC_t_, area under the plasma concentration–time curve from time zero to the last measured time point; AUC_∞_, area under the plasma concentration–time curve from time zero extrapolated to infinity; C_max_, maximum plasma concentration; t_max_, time to maximum plasma concentration; t_1/2_, elimination half-life.

### Efficacy

**Figure 2.**
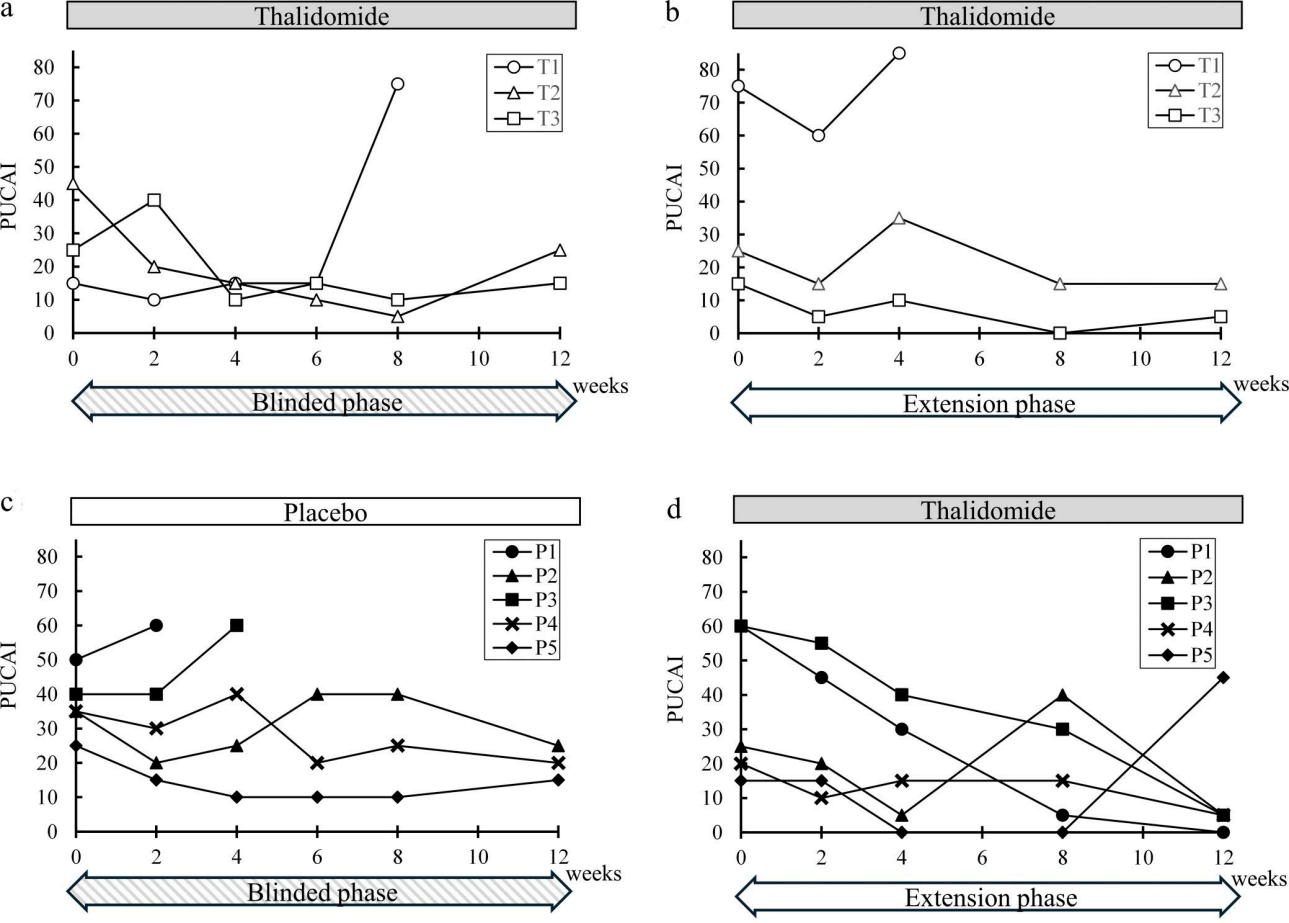
**PUCAI scores of patients in the blinded phase and the extension phase. (a and b)** In the thalidomide group, the patients were treated with thalidomide in the blinded phase (a) and the extension phase (b). The final markers of each line in a and the initial markers of each line in b are at the same time point, corresponding to the transition to the extension phase in the trial. **(c and d)** In the placebo group, the patients were treated with a placebo in the blinded phase (c) and with thalidomide in the extension phase (d). The final markers of each line in c and the initial markers of each line in d are at the same time point, corresponding to the transition to the extension phase in the trial.

At the end of the blinded phase (or at discontinuation), the primary endpoint (PUCAI decrease ≥20 points or score <10) was achieved by one of three patients (0.33; 95% confidence interval [CI], 0.01–0.91) in the thalidomide group and by none of five (0; 95% CI, 0–0.52) in the placebo group (Table 4 and Fig. 2). At the end of the extension phase (or at discontinuation), five of eight patients (0.63; 95% CI, 0.25–0.92) achieved the primary endpoint; of these, one had previously received thalidomide without meeting the endpoint and four had received placebo in the blinded phase (Fig. S1 b and Fig. S2, b–e). The remaining three patients (T1, T2, and P5) who did not achieve the primary endpoint in the extension phase all had concomitant bacterial infections.

**Table 4.** Efficacy of thalidomide for CGD-IBD in the trial

Category	Definition	Period	Group	Categorization	Frequency	Achievement rate	95% CIs (lower, upper limits)	Difference in achievement rate	95% CIs of the difference (lower, upper limits)	Achievement rate ratio	95% CIs of the ratio (lower, upper limits)
Primary endpoint	PUCAI decrease ≥20 points or score <10	Blinded phase	Thalidomide (*n* = 3)	Achieved	1	0.33	0.01, 0.91	0.33	−0.37, 0.91	∞	0.13, ∞
				Not achieved	2						
			Placebo (*n* = 5)	Achieved	0	0	0, 0.52				
				Not achieved	5						
		Extension phase	All (*n* = 8)	Achieved	5	0.63	0.25, 0.92	–	–	–	–
				Not achieved	3						
Secondary endpoint	PUCAI <10	Blinded phase	Thalidomide (*n* = 3)	Achieved	0	0	0, 0.71	–	–	–	–
				Not achieved	3						
			Placebo (*n* = 5)	Achieved	0	0	0, 0.52				
				Not achieved	5						
		Extension phase	All (*n* = 8)	Achieved	5	0.63	0.25, 0.92	–	–	–	–
				Not achieved	3						
	PGA with ≥1 grade improvement	Blinded phase	Thalidomide (*n* = 3)	Achieved	1	0.33	0.01, 0.91	0.13	−0.54, 0.77	–	–
				Not achieved	2						
			Placebo (*n* = 5)	Achieved	1	0.20	0.01, 0.72				
				Not achieved	4						
		Extension phase	All (*n* = 8)	Achieved	5	0.63	0.25, 0.92	–	–	–	–
				Not achieved	3						

PUCAI, Pediatric Ulcerative Colitis Activity Index; PGA, Physician Global Assessment, ∞, infinity.

For secondary endpoints, no patients achieved PUCAI remission in the blinded phase. PGA with ≥1 grade improvement was observed in one of three patients (0.33; 95% CI, 0.01–0.91) in the thalidomide group and one of five (0.20; 95% CI, 0.01–0.72) in the placebo group. In the extension phase, five of eight patients (0.63; 95% CI, 0.25–0.92) achieved both PUCAI remission and PGA improvement (Fig. S1 b and Fig. S2, b–e).

**Figure 3.**
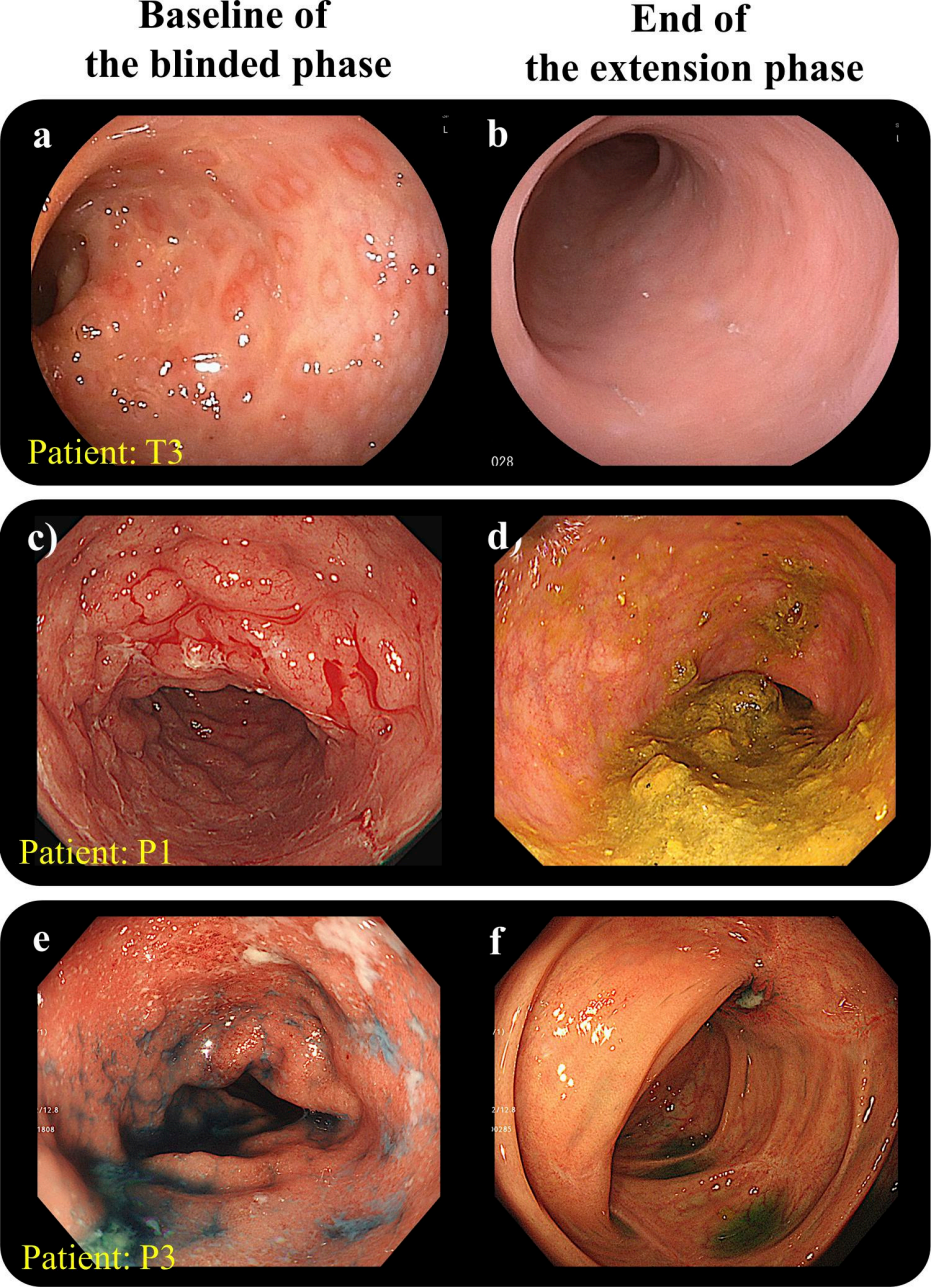
**Improvement in colonoscopy findings after thalidomide administration. (a and b)** In T3, who achieved remission at the end of the extension phase, lymphoid hyperplasia with red halos was prominent in the distal sigmoid colon before the trial (a). No red halos were noted after the extension phase (b). **(c and d)** In P1, who discontinued the blinded phase and achieved remission at the end of the extension phase, edematous nodular mucosa with whitish exudate was noted in the rectum before the trial (c). Vascular marking was restored with some erosions after the extension phase (d). **(e and f)** In P3, who discontinued the blinded phase and achieved remission at the end of the extension phase, diffuse edematous granular mucosa was noted with multiple ulcers in the proximal sigmoid colon before the trial (e). Vascular marking was restored with scarring and a few ulcers after the extension phase (f).

**Figure S3.**
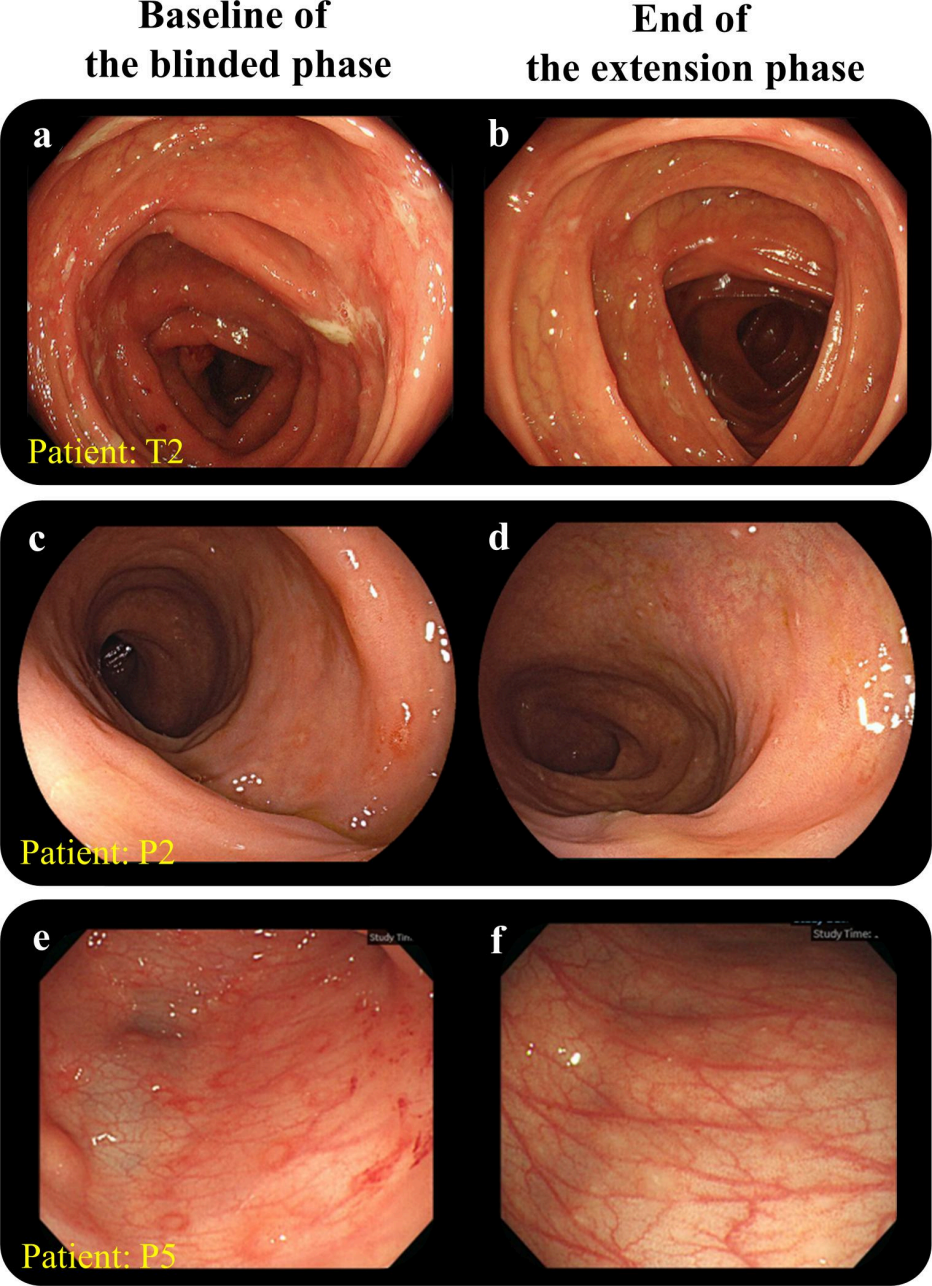
**Improvement in colonoscopy findings after thalidomide administration. (a and b)** In patient T2, erythematous edematous mucosa with erosions and small ulcers was observed in the ascending colon before the trial (a). After the extension phase, vascular markings were largely restored, with some residual erosions (b). **(c and d)** In patient P2, several rectal erosions were noted before the trial (c). These findings showed relative improvement after the extension phase (d). **(e and f)** In patient P5, prominent lymphoid hyperplasia with red halos was observed in the descending colon before the trial (e). After the extension phase, the red halos were no longer evident (f).

Seven of eight patients underwent colonoscopy; patient P4 in the placebo group, in remission at the end of the extension phase, did not. For patient T1 in the thalidomide group who discontinued during the blinded phase due to rapid PUCAI worsening, colonoscopy showed mucosal lesion exacerbation. In three patients (patient P1 and patient P3 in the placebo group who discontinued the blinded phase and entered the extension phase, and patient T3 in the thalidomide group in remission during the extension phase), marked mucosal improvement was observed at the end of thalidomide administration (Fig. 3). In the other three patients (T2, P2, and P5), similar qualitative improvements were also observed after the extension phase, although the degree of change was more subtle because these cases predominantly had mild mucosal inflammatory changes at baseline (Fig. S3).

### Safety

During the blinded phase, adverse events were reported in three patients per group; in the extension phase, adverse events occurred in seven patients, mostly grade ≤3 and primarily grade 1 or 2. Adverse events considered related to thalidomide included liver dysfunction, muscular pain, drowsiness, and erythema in the blinded phase, and infectious enteritis in the extension phase. Drowsiness was reported in one adult patient in the thalidomide group, but not in any other patients. No thalidomide-specific peripheral neuropathy or deep vein thrombosis occurred (Table 5).

**Table 5.** Adverse events in the blinded phase and the extension phase

System organ class	Adverse events	Blinded phase		Extension phase
		Thalidomide (*n* = 3)	Placebo (*n* = 5)	All (*n* = 8)
Gastrointestinal disorders	Gastritis	1 (33.3%)	1 (20.0%)	1 (12.5%)
	Stomatitis	0	1 (20.0%)	0
	Diarrhea	0	0	1 (12.5%)
	Hemorrhoid	0	0	1 (12.5%)
General disorders and administration site conditions	Fever	0	1 (20.0%)	1 (12.5%)
Infections	Upper respiratory tract viral infection	1 (33.3%)	0	0
	Viral gastroenteritis	1 (33.3%)	0	0
	Aspergillosis	1 (33.3%)	0	0
	Otitis media	1 (33.3%)	0	0
	Pustular eczema	1 (33.3%)	0	0
	Thymic abscess	0	1 (20.0%)	0
	Respiratory syncytial virus infection	0	0	1 (12.5%)
	*Clostridium difficile* infection	0	0	1 (12.5%)
	Pharyngitis	0	0	1 (12.5%)
	Balanoposthitis	0	0	1 (12.5%)
	Infectious enteritis	0	0	1 (12.5%)
	Esophageal candidiasis	0	0	1 (12.5%)
	Perirectal abscess	0	0	1 (12.5%)
Hepatobiliary disorders	Liver dysfunction	3 (100.0%)	0	0
Musculoskeletal and connective tissue disorders	Muscular pain	1 (33.3%)	0	0
	Backache	0	0	1 (12.5%)
Blood and lymphatic system disorders	Anemia	0	2 (40.0%)	3 (37.5%)
	Lymphadenitis	1 (33.3%)	0	1 (12.5%)
Injury, poisoning, and procedural complications	Arthropod sting	1 (33.3%)	0	0
	Insect bite and sting	1 (33.3%)	0	0
	Redness of catheterization skin	0	0	1 (12.5%)
Nervous system disorder	Drowsiness	1 (33.3%)	0	0
	Febrile convulsion	1 (33.3%)	0	0
Skin and subcutaneous tissue disorders	Erythema	1 (33.3%)	0	1 (12.5%)
	Rash	1 (33.3%)	1 (20.0%)	1 (12.5%)
	Urticaria	1 (33.3%)	0	0
Immune system disorders	Anaphylactic reaction	0	1 (20.0%)	0
	Allergy	0	0	1 (12.5%)
Investigations	Coagulation disorder	1 (33.3%)	0	0
	C-reactive protein increase	1 (33.3%)	​	​
	Aspartate aminotransferase increase	0	0	2 (25.0%)
	Alanine aminotransferase increase	0	0	1 (12.5%)

In the blinded phase, two patients on thalidomide experienced three serious adverse events, namely, lymphadenitis and C-reactive protein increase due to bacterial infection (all CGD-related infections, Table 5), and one febrile convulsion. One placebo patient had a serious adverse event of thymic abscess (CGD-related infection). In the extension phase, one thalidomide patient from the blinded phase developed a perirectal abscess. Two placebo patients from the blinded phase experienced three serious adverse events, namely, lymphadenitis and infectious enteritis (CGD-related infections) and one hemorrhagic anemia. All serious adverse events resolved and were considered unrelated to study treatment.

12 CGD-related infections occurred within 24 wk after starting the investigational drug, including pulmonary aspergillosis, lymphadenitis, and esophageal candidiasis. The posttreatment incidence rates were 9.78 (95% CI, 3.59–21.30) per patient-year in the thalidomide group and 7.35 (95% CI, 2.70–16.01) per patient-year in the placebo group (Table 6). No clear differences were observed compared with pretreatment rates in either group. In the extension phase, the posttreatment incidence rate was 3.51 (95% CI, 1.29–7.63) per patient-year among eight patients who received thalidomide, which was not higher than the pretreatment incidence rate.

**Table 6.** Incidence of infection related to CGD

Group	Period	Frequency	Incidence rate	95% CIs (lower, upper limits)	Difference in incidence rate	95% CIs of the difference (lower, upper limits)	Incidence rate ratio	95% CIs of the ratio (lower, upper limits)
Thalidomide (*n* = 3)	24 wk before administration	7	5.07	2.04, 10.45	4.71	−3.97, 13.39	1.93	0.54, 6.70
	24 wk after administration	6	9.78	3.59, 21.30				
Placebo (*n* = 5)	24 wk before administration	12	5.22	2.70, 9.12	2.14	−4.45, 8.72	1.41	0.43, 4.06
	24 wk after administration	6	7.35	2.70, 16.01				
All (*n* = 8)	Extension phase	6	3.51	1.29, 7.63	–	–	–	–

Infections in the thalidomide group and placebo group were assessed by extracting data from medical records prior to initiation of the investigational drug and aggregating data during the blinded phase (12 wk) and the subsequent extension phase (12 wk). 95% CIs, 95% confidence intervals.

## Discussion

This clinical study was conducted to generate foundational data to support future clinical study evaluating the efficacy of thalidomide in CGD-IBD patients. This randomized, placebo-controlled trial, the first to investigate thalidomide in patients with CGD-IBD, was designed to balance feasibility in an ultrarare disease setting with scientific rigor by incorporating a prespecified efficacy criterion to guide interpretation. In the blinded phase, this criterion was met, as one of three patients in the thalidomide group achieved the primary endpoint by PUCAI improvement compared with none of five in the placebo group. In the extension phase, five of eight patients achieved the primary endpoint, as well as the secondary endpoints of PUCAI remission and PGA improvement, with marked mucosal improvement on colonoscopy. The incidence of CGD-related infections was comparable before and after treatment and between groups, and no grade 4–5 or other thalidomide-related serious adverse events occurred, suggesting that thalidomide was well tolerated without increasing infection susceptibility. Although further accumulation of cases is warranted to clarify the impact of thalidomide on susceptibility to infections, the balance between the efficacy and safety of thalidomide treatment appeared acceptable, particularly given the unmet need for anti-inflammatory options that do not heighten infection risk in CGD-IBD.

The present findings are consistent with previous reports of anti-inflammatory effects of thalidomide in chronic inflammatory diseases such as Behçet’s disease, rheumatoid arthritis, Crohn’s disease, and ulcerative colitis (20, 21, 27, 28, 29, 30). Case reports of CGD-IBD have described symptom improvement with thalidomide combined with corticosteroids or immunosuppressants (17, 25, 26), but the efficacy of thalidomide monotherapy had not been evaluated. Our trial indicates, for the first time, that thalidomide can improve CGD-IBD symptoms without concomitant immunosuppressive therapy. Although the precise mechanisms remain unclear, thalidomide attenuates TNF-α–induced inflammation without affecting lipopolysaccharide-driven cytokine production in monocytes from both healthy individuals and CGD patients (17, 18). Our results therefore align with and extend these prior mechanistic and clinical observations.

Although the association between infections and disease activity of CGD-IBD remains unclear, in the present trial, five patients experienced infections concomitant with an increase in the PUCAI. In CGD, microbial stimulation induces dysregulated inflammatory cytokine production, including TNF-α, which promotes macrophage activation and granuloma formation (31). The pathogenesis of CGD-IBD has also been linked to inflammasome activation and defective autophagy under elevated IL-1β associated with reactive oxygen species deficiency in mouse models (11, 32). Unlike general IBD, infection-related cytokines in CGD may activate intestinal macrophages via systemic circulation, exacerbating intestinal inflammation. However, no reports, including the present trial, have shown PUCAI reduction due to infections in ulcerative colitis or CGD-IBD. Therefore, it is unlikely that the efficacy of thalidomide was overestimated.

A strength of this trial lies in its design tailored to patients with congenital immunodeficiency and their families. Because approximately half of the patients would receive placebo during the blinded phase, predefined discontinuation criteria were incorporated to ensure that patients with worsening or refractory symptoms could transition to the extension phase and receive active treatment. In fact, all patients opted to enter the extension phase after the blinded phase. Moreover, the novel orally disintegrating tablet (ODT) formulation of thalidomide enabled administration even in toddlers and ensured 100% adherence. As thalidomide is available only in capsule formulations, administration to infants is challenging; indeed, only a few cases have reported its use in infants around 1 year of age (33, 34, 35). Previously, pharmacokinetics for thalidomide in children were unclear, but pharmacokinetic parameters in three patients, including two pediatric patients and one adult patient, were consistent with adult profiles (36, 37, 38), confirming the appropriateness of the ODT formulation. Clinically, the ODT minimized handling and exposure risks of this potentially teratogenic drug for caregivers and healthcare providers. Notably, the trial incorporated patient and public involvement (39): a patients’ association reviewed study documents, and a family-inclusive risk management program was implemented, reflecting patient-centered strategies not yet widely adopted in Japan, which enhanced both ethics and feasibility.

This study has several limitations. First, the major limitation was the very small sample size (*n* = 8) in this trial for an ultrarare disease. The imbalance in allocation (three patients in the thalidomide group and five in the placebo group), resulting from stratified block randomization, may have further limited the precision of the findings. Due to the sample size of this study, a definitive assessment of efficacy would be difficult; however, the therapeutic effect observed with thalidomide for CGD-IBD (1/3 patients in the blinded phase and 5/8 in the extension phase) was consistent with previous trials of refractory IBD, which reported very low numbers needed to treat for clinical remission (1.5 in ulcerative colitis [18] and 2.86 in Crohn’s disease [19]). Second, both PUCAI and PGA are physician-assessed measures, and there are no validated biomarkers for CGD-IBD. However, the randomized, blinded design minimized potential bias, and the findings were consistent across primary and secondary endpoints, supported by exploratory colonoscopy findings. Third, the treatment period was limited to 24 wk; therefore, long-term efficacy and safety, particularly the risks associated with prolonged thalidomide use, including neuropathy and thrombosis, remain to be clarified. Previous trials in pediatric ulcerative colitis and Crohn’s disease reported peripheral neuropathy as a serious adverse event (20, 21), and an observational study in adults with intractable Crohn’s disease demonstrated increased risks of peripheral neuropathy and deep vein thrombosis with extended therapy (40). In this study, nerve conduction studies were performed repeatedly to enable the early detection of neuropathy, even in pediatric patients who may have difficulty articulating subjective symptoms (Table S2), and no such events occurred in our trial. CGD patients may carry comparable risks, and CGD-IBD may relapse. Therefore, in the long term, thalidomide may be positioned as a bridging therapy leading to curative treatment with hematopoietic stem cell transplantation in CGD.

In conclusion, although a definitive assessment of efficacy and safety would be difficult given the small sample size of this study, this trial indicated that thalidomide ODT met the prespecified criterion for interpreting the study’s efficacy results, with an acceptable safety profile in patients aged ≥1 year with CGD-IBD. Thalidomide may represent a promising therapeutic option for controlling intestinal inflammation without increasing susceptibility to infection, and further clinical studies are warranted to clarify its role in the management of CGD-IBD.

## Materials and methods

### Study design

**Figure 4.**
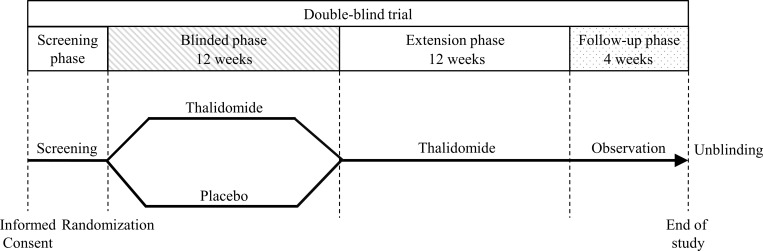
**Study design.** The patients were randomly assigned to a 12-wk treatment of thalidomide or placebo (blinded phase) followed by a 12-wk extension phase (thalidomide administration). The patients were visited at 4 wk after the treatment (follow-up phase).

We conducted a multicenter, randomized, double-blind phase II trial of thalidomide for CGD-IBD at seven tertiary care institutions in Japan with expertise in pediatric immunodeficiency. Patients were randomized to thalidomide or placebo for 12 wk in the blinded phase, followed by a 12-wk extension phase in which those who reconsented to active treatment received thalidomide (Fig. 4 and Table S2). Given the rarity of CGD-IBD, the trial prioritized feasibility over confirmatory hypothesis testing and was therefore conducted with a small sample size, without aiming to demonstrate statistical superiority to placebo. Instead, prespecified criteria for interpreting the results were set to guide subsequent decision-making on the role of thalidomide. As this was the first trial of thalidomide in CGD-IBD and the natural history of the disease remains unclear, a double-blind, placebo-controlled, parallel-group design, rather than an external control, was considered necessary (41, 42). This trial design was advised by the Pharmaceuticals and Medical Devices Agency, Japan’s regulatory authority, and was approved on November 28, 2016.

The study complied with Good Clinical Practice and the principles of the Declaration of Helsinki, and was approved by the institutional review boards of all participating institutions. Written informed consent was obtained from all adult patients and from parents or legal guardians of pediatric patients, with assent from participants aged ≥7 years. The trial was registered in the UMIN Clinical Trials Registry (UMIN000029324) and the Japan Registry of Clinical Trials (jRCT2031200236) prior to enrollment of the first patient. Data supporting the findings of this study are provided in this article and its supplementary materials.

All patients and families were informed of potential adverse effects, including teratogenicity, contraception requirements, and exposure risks. In Japan, thalidomide is manufactured and marketed by Fujimoto Pharmaceutical Corporation, which also provides the Thalidomide Education and Risk Management System (TERMS) to minimize teratogenic risk. A family-inclusive risk management program was implemented in consultation with Ishizue (Public Interest Incorporated Foundation of Thalidomide Victims) in Japan, with reference to the TERMS; the association also reviewed the protocol, informed consent/assent forms, and the explanatory brochure during study planning.

### Study population

CGD was diagnosed by the absence of neutrophil superoxide production and confirmed by genetic testing. Eligible patients were ≥1 year old with CGD-IBD meeting all the following criteria (1): persistent enteritis symptoms (abdominal pain, diarrhea, or hematochezia) (2); exclusion of other causes of chronic enteritis (infectious, drug-induced, or allergic); and (3) supportive histopathology on colonic biopsy (inflammatory infiltrates, granulomas, or foamy macrophages).

Disease severity of CGD-IBD was classified using the PUCAI (20). The PUCAI ranges from 0 to 85, with remission defined as 0≤ PUCAI <10, mild as 10≤ PUCAI <35, moderate as 35≤ PUCAI <65, and severe as 65≤ PUCAI, based on previous IBD studies (20, 43). For enrollment, patients were eligible if they had moderate disease or mild disease accompanied by hematochezia or ≥6 stools daily.

Exclusion criteria for enrollment were the following: severe hypersensitivity or allergy, macrophage activation syndrome, pregnancy or breastfeeding, prior thalidomide exposure, systemic or rectal steroids within 4 wk, immunomodulators within 8 wk, or prior biologics (anti-TNF-α or anti-IL-1).

### Study treatment

An ODT formulation of thalidomide was developed for this trial to enable administration in children from 1 year of age. Appropriate nonclinical studies (toxicology, stability, and dissolution) and a phase I bioequivalence study were completed prior to the trial. Fujimoto Pharmaceutical Corporation manufactured and supplied the ODT of thalidomide and matching placebo (5, 10, and 25 mg), identical in appearance and packaging.

Patients judged eligible at screening were randomized to thalidomide or placebo in an approximate 1:1 ratio using the permutation block method stratified by CGD-IBD severity at screening. Patients, guardians, treating physicians, outcome assessors, and all investigators, including the biostatistician, were blinded to group assignments. Corresponding drug numbers were preassigned and affixed to study drugs by EPS Corporation; randomization and allocation were subsequently managed via the electronic data capture and registration system (Viedoc Japan K.K.).

During the 12-wk blinded phase, patients received thalidomide or placebo as an ODT once daily at bedtime, with the first dose administered 2 h after breakfast. The investigators explained to patients and families that thalidomide ODT is highly susceptible to moisture. Treatment was initiated at 2 mg/kg/day and titrated to 1–3 mg/kg/day (maximum 200 mg/day) according to disease activity and adverse events. If the PUCAI increased ≥20 points from baseline, the dose could be increased to 3 mg/kg. The dose was increased to 3 mg/kg if, after 2 wk at 2 mg/kg, PUCAI remained ≥10 and the reduction from baseline was <20 points. Treatment failure with relapse was defined as a ≥20-point increase in PUCAI from baseline, and treatment was discontinued if the investigator judged it necessary. If adverse events occurred, dosing was maintained, reduced, interrupted, or discontinued according to the severity of adverse events (44). Because the study population included pediatric patients, including young children, the trial prioritized patient safety, with disease activity closely monitored based on clinical symptoms and the PUCAI.

Blood samples for plasma thalidomide concentrations were collected at 1, 2, 4, 10, and 24 h after the first dose, and 24 h after dosing at wk 6 and 12. Plasma levels were quantified using validated liquid chromatography–tandem mass spectrometry, and pharmacokinetic parameters were calculated. Investigators, patients, and their families remained blinded to centrally monitored pharmacokinetic data throughout the trial.

At the end of the blinded phase, patients could either initiate conventional immunosuppressive therapy or proceed to a 12-wk extension phase of once-daily thalidomide, followed by a 4-wk follow-up (Fig. 1). To maintain blinding, group assignments were unblinded after all patients had completed follow-up. In the extension phase, thalidomide was administered once daily at bedtime, starting at 2 mg/kg/day in patients without safety concerns during the blinded phase, and subsequent dosing was managed as in the blinded phase.

Concomitant use of corticosteroids, immunosuppressants, and biologics, and most antimicrobials was prohibited, except for trimethoprim/sulfamethoxazole, itraconazole, and voriconazole.

### Study outcomes

The schedule of study visits and data collection, including efficacy and safety evaluation, is provided in Table S2. Efficacy was evaluated using PUCAI, validated in pediatric ulcerative colitis (43), and applicable to CGD-IBD (45), with a ≥20-point reduction defined as clinical response based on prior validation and recommendations (43). The primary endpoint was the proportion of patients who achieved either a reduction of ≥20 points from baseline or remission (PUCAI <10) at the end of each phase (or at discontinuation). Secondary endpoints were the proportions of patients in remission (PUCAI <10) and those with an improvement of ≥1 grade in PGA at the end of each phase (or at discontinuation). PGA, which has been used as a measure of disease severity in ulcerative colitis, classified patients as normal, mild, moderate, or severe based on daily abdominal discomfort, overall well-being, performance status, and physical findings assessed by investigators (46). As an exploratory evaluation, colonoscopy was performed in patients judged capable of undergoing the procedure at baseline of the blind phase and at the end of the extension phase.

Safety was assessed by adverse events collected through passive surveillance (patient self-reports, physician examination, and clinically relevant laboratory or vital sign changes) and recorded at scheduled visits, graded according to the National Cancer Institute Common Terminology Criteria for Adverse Events (47), from grade 1 (mild) to grade 5 (death related to the event). As a key safety evaluation, the incidence of CGD-related infections was assessed during the 24 wk before initiation and the 24 wk after completion of thalidomide treatment. CGD-related infections included bacterial and fungal infections but excluded viral infections. Data on infections in the 24 wk before treatment were obtained retrospectively from medical records.

### Statistical analysis

Considering the rarity of CGD-IBD and a feasibility survey showing that only six eligible patients were identified across participating institutions over 3 years, the sample size was set at eight. The protocol prespecified criterion for interpreting the study results as follows: thalidomide would be considered effective if more patients in the thalidomide group achieved the primary endpoint than in the placebo group during the blinded phase. For efficacy evaluation, the primary and secondary endpoints in each group were estimated with 95% CIs using the Clopper–Pearson method. Efficacy analysis included all patients who received at least one dose of the investigational drug and had the necessary data, whereas safety analysis included all patients who received at least one dose of the investigational drug. For safety evaluation, adverse events were summarized as frequencies and percentages. The incidence rate of CGD-related infections was estimated for the 24 wk before and after treatment in the blinded phase, as well as during the extension phase, with 95% CIs calculated based on the Poisson distribution. The difference between the incidence rates, together with their 95% CIs, was also calculated. All analyses were performed using SAS version 14 (SAS Institute Inc.). Missing data were not imputed, and no interim analyses were planned.

### Declaration of generative AI and AI-assisted technologies in the manuscript preparation process

During the preparation of this work, the authors used Grammarly, DeepL, and ChatGPT in order to check the accuracy and to improve readability of the English text. After using these tools, the authors carefully reviewed and edited the content, and the final draft was further reviewed by a native English-speaking medical editor. The authors take full responsibility for the content of the published article.

### Online supplemental material

Supplementary information includes additional data on PUCAI (Table S1, Fig. S1, and Fig. S2) and PGA (Table S1), methodological details (Table S2), and endoscopic findings (Fig. S3).

## Supplementary Material

Table S1 shows PUCAI and PGA for individual patients in the trial.

Table S2 shows schedule for the study visits and data collection.

## Data Availability

The data underlying this study are not publicly available due to patient privacy issues.

## References

[1] Prince, B.T., B.K. Thielen, K.W. Williams, E.S. Kellner, D.E. Arnold, W. Cosme-Blanco, M.T. Redmond, N.L. Hartog, H.J. Chong, and S.M. Holland. 2020. Geographic variability and Pathogen-specific considerations in the diagnosis and management of chronic granulomatous disease. Pediatric Health Med. Ther. 11:257–268. 10.2147/phmt.s254253 32801991 PMC7383027

[2] Nunoi, H. 2007. [Two breakthroughs in CGD studies]. Nihon Rinsho Meneki Gakkai Kaishi. 30:1–10. 10.2177/jsci.30.1 17332699

[3] Marciano, B.E., C. Spalding, A. Fitzgerald, D. Mann, T. Brown, S. Osgood, L. Yockey, D.N. Darnell, L. Barnhart, J. Daub, . 2015. Common severe infections in chronic granulomatous disease. Clin. Infect. Dis. 60:1176–1183. 10.1093/cid/ciu1154 25537876 PMC4400412

[4] Arnold, D.E., and J.R. Heimall. 2017. A review of chronic granulomatous disease. Adv. Ther. 34:2543–2557. 10.1007/s12325-017-0636-2 29168144 PMC5709447

[5] Falcone, E.L., and S.M. Holland. 2019. Gastrointestinal complications in chronic granulomatous disease. Methods Mol. Biol. 1982:573–586. 10.1007/978-1-4939-9424-3_34 31172496

[6] Marciano, B.E., S.D. Rosenzweig, D.E. Kleiner, V.L. Anderson, D.N. Darnell, S. Anaya-O'Brien, D.M. Hilligoss, H.L. Malech, J.I. Gallin, and S.M. Holland. 2004. Gastrointestinal involvement in chronic granulomatous disease. Pediatrics. 114:462–468. 10.1542/peds.114.2.462 15286231

[7] Schappi, M.G., N.J. Klein, K.J. Lindley, D. Rampling, V.V. Smith, D. Goldblatt, and P.J. Milla. 2003. The nature of colitis in chronic granulomatous disease. J. Pediatr. Gastroenterol. Nutr. 36:623–631. 10.1002/j.1536-4801.2003.tb08083.x 12717086

[8] Alimchandani, M., J.P. Lai, P.P. Aung, S. Khangura, N. Kamal, J.I. Gallin, S.M. Holland, H.L. Malech, T. Heller, M. Miettinen, and M.M. Quezado. 2013. Gastrointestinal histopathology in chronic granulomatous disease: A study of 87 patients. Am. J. Surg. Pathol. 37:1365–1372. 10.1097/pas.0b013e318297427d 23887163 PMC3787986

[9] Marks, D.J.B., K. Miyagi, F.Z. Rahman, M. Novelli, S.L. Bloom, and A.W. Segal. 2009. Inflammatory bowel disease in CGD reproduces the clinicopathological features of Crohn’s disease. Am. J. Gastroenterol. 104:117–124. 10.1038/ajg.2008.72 19098859

[10] Zerbe, C.S., and S.M. Holland. 2024. Functional neutrophil disorders: Chronic granulomatous disease and beyond. Immunol. Rev. 322:71–80. 10.1111/imr.13308 38429865 PMC10950525

[11] de Luca, A., S.P. Smeekens, A. Casagrande, R. Iannitti, K.L. Conway, M.S. Gresnigt, J. Begun, T.S. Plantinga, L.A.B. Joosten, J.W.M. van der Meer, . 2014. IL-1 receptor blockade restores autophagy and reduces inflammation in chronic granulomatous disease in mice and in humans. Proc. Natl. Acad. Sci. USA. 111:3526–3531. 10.1073/pnas.1322831111 24550444 PMC3948220

[12] Hahn, K.J., N. Ho, L. Yockey, S. Kreuzberg, J. Daub, A. Rump, B.E. Marciano, M. Quezado, H.L. Malech, S.M. Holland, . 2015. Treatment with Anakinra, a recombinant IL-1 receptor antagonist, unlikely to induce lasting remission in patients with CGD colitis. Am. J. Gastroenterol. 110:938–939. 10.1038/ajg.2015.135 26052777

[13] Conrad, A., B. Neven, N. Mahlaoui, F. Suarez, H. Sokol, F.M. Ruemmele, C. Rouzaud, D. Moshous, O. Lortholary, S. Blanche, and F. Lanternier. 2021. Infections in patients with chronic granulomatous disease treated with tumor necrosis factor alpha blockers for inflammatory complications. J. Clin. Immunol. 41:185–193. 10.1007/s10875-020-00901-8 33150502

[14] Uzel, G., J.S. Orange, N. Poliak, B.E. Marciano, T. Heller, and S.M. Holland. 2010. Complications of tumor necrosis factor-alpha blockade in chronic granulomatous disease-related colitis. Clin. Infect. Dis. 51:1429–1434. 10.1086/657308 21058909 PMC3106244

[15] Bhattacharya, S., B.E. Marciano, H.L. Malech, M. Quezado, S.M. Holland, S.S. De Ravin, C.S. Zerbe, and T. Heller. 2022. Safety and efficacy of Ustekinumab in the inflammatory bowel disease of chronic granulomatous disease. Clin. Gastroenterol. Hepatol. 20:461–464.e2. 10.1016/j.cgh.2021.03.039 33813069

[16] Butte, M.J., K.T. Park, and D.B. Lewis. 2016. Treatment of CGD-associated colitis with the IL-23 blocker Ustekinumab. J. Clin. Immunol. 36:619–620. 10.1007/s10875-016-0318-x 27465505 PMC5018915

[17] Kawai, T., N. Watanabe, M. Yokoyama, K. Arai, S. Oana, S. Harayama, K. Yasui, T. Oh-ishi, and M. Onodera. 2013. Thalidomide attenuates excessive inflammation without interrupting lipopolysaccharide-driven inflammatory cytokine production in chronic granulomatous disease. Clin. Immunol. 147:122–128. 10.1016/j.clim.2013.03.004 23583898

[18] Majumdar, S., B. Lamothe, and B.B. Aggarwal. 2002. Thalidomide suppresses NF-kappa B activation induced by TNF and H_2_O_2_, but not that activated by ceramide, lipopolysaccharides, or phorbol ester. J. Immunol. 168:2644–2651. 10.4049/jimmunol.168.6.2644 11884428

[19] Peng, X., Z.W. Lin, M. Zhang, J.Y. Yao, J.Z. Zhao, P.J. Hu, Q. Cao, and M. Zhi. 2022. The efficacy and safety of thalidomide in the treatment of refractory Crohn’s disease in adults: A double-center, double-blind, randomized-controlled trial. Gastroenterol. Rep (Oxf). 10:goac052. 10.1093/gastro/goac052 36284737 PMC9583847

[20] Lazzerini, M., S. Martelossi, G. Magazzu, S. Pellegrino, M.C. Lucanto, A. Barabino, A. Calvi, S. Arrigo, P. Lionetti, M. Lorusso, . 2015. Effect of Thalidomide on clinical remission in children and adolescents with ulcerative colitis refractory to other immunosuppressives: Pilot randomized clinical trial. Inflamm. Bowel Dis. 21:1739–1749. 10.1097/mib.0000000000000437 26185909

[21] Lazzerini, M., S. Martelossi, G. Magazzu, S. Pellegrino, M.C. Lucanto, A. Barabino, A. Calvi, S. Arrigo, P. Lionetti, M. Lorusso, . 2013. Effect of thalidomide on clinical remission in children and adolescents with refractory Crohn disease: A randomized clinical trial. JAMA. 310:2164–2173. 10.1001/jama.2013.280777 24281461

[22] Sampaio, E.P., E.N. Sarno, R. Galilly, Z.A. Cohn, and G. Kaplan. 1991. Thalidomide selectively inhibits tumor necrosis factor alpha production by stimulated human monocytes. J. Exp. Med. 173:699–703. 10.1084/jem.173.3.699 1997652 PMC2118820

[23] D'Amato, R.J., M.S. Loughnan, E. Flynn, and J. Folkman. 1994. Thalidomide is an inhibitor of angiogenesis. Proc. Natl. Acad. Sci. USA. 91:4082–4085. 10.1073/pnas.91.9.4082 7513432 PMC43727

[24] Corral, L.G., P.A. Haslett, G.W. Muller, R. Chen, L.M. Wong, C.J. Ocampo, R.T. Patterson, D.I. Stirling, and G. Kaplan. 1999. Differential cytokine modulation and T cell activation by two distinct classes of thalidomide analogues that are potent inhibitors of TNF-alpha. J. Immunol. 163:380–386. 10.4049/jimmunol.163.1.380 10384139

[25] Noel, N., N. Mahlaoui, S. Blanche, F. Suarez, H. Coignard-Biehler, I. Durieu, P. Godeberge, H. Sokol, E. Catherinot, S. Poiree, . 2013. Efficacy and safety of thalidomide in patients with inflammatory manifestations of chronic granulomatous disease: A retrospective case series. J. Allergy Clin. Immunol. 132:997–1000.e1-4. 10.1016/j.jaci.2013.04.059 23791514

[26] Sokol, H., F. Suarez, T. Meatchi, G. Malamut, M.A. Pocidalo, S. Blanche, C. Cellier, and O. Hermine. 2009. Thalidomide as a treatment for refractory CGD colitis. Am. J. Gastroenterol. 104:1069. 10.1038/ajg.2009.56 19293783

[27] Eski, M., I. Sahin, M. Sengezer, M. Serdar, and A. Ifran. 2008. Thalidomide decreases the plasma levels of IL-1 and TNF following burn injury: Is it a new drug for modulation of systemic inflammatory response. Burns. 34:104–108. 10.1016/j.burns.2007.01.007 17618052

[28] Lazzerini, M., S. Martelossi, F. Marchetti, A. Scabar, F. Bradaschia, L. Ronfani, and A. Ventura. 2007. Efficacy and safety of thalidomide in children and young adults with intractable inflammatory bowel disease: Long-term results. Aliment. Pharmacol. Ther. 25:419–427. 10.1111/j.1365-2036.2006.03211.x 17269997

[29] Meierhofer, C., and C.J. Wiedermann. 2003. New insights into the pharmacological and toxicological effects of thalidomide. Curr. Opin. Drug Discov. Devel. 6:92–99. 12613280

[30] Lazzerini, M., V. Villanacci, M.C. Pellegrin, S. Martelossi, G. Magazzu, S. Pellegrino, M.C. Lucanto, A. Barabino, A. Calvi, S. Arrigo, . 2017. Endoscopic and histologic healing in children with inflammatory bowel diseases treated with thalidomide. Clin. Gastroenterol. Hepatol. 15:1382–1389.e1. 10.1016/j.cgh.2017.02.029 28286192

[31] Kuijpers, T., and R. Lutter. 2012. Inflammation and repeated infections in CGD: Two sides of a coin. Cell Mol. Life Sci. 69:7–15. 10.1007/s00018-011-0834-z 22083605 PMC3249194

[32] Huang, C., S.S. De Ravin, A.R. Paul, T. Heller, N. Ho, L. Wu Datta, C.S. Zerbe, B.E. Marciano, D.B. Kuhns, H.A. Kader, . 2016. Genetic risk for inflammatory bowel disease is a determinant of Crohn’s disease development in chronic granulomatous disease. Inflamm. Bowel Dis. 22:2794–2801. 10.1097/mib.0000000000000966 27861181 PMC5303573

[33] van Toorn, R., R.S. Solomons, J.A. Seddon, and J.F. Schoeman. 2021. Thalidomide use for complicated central nervous system tuberculosis in children: Insights from an observational cohort. Clin. Infect. Dis. 72:e136–e145. 10.1093/cid/ciaa1826 33283220

[34] Frei-Jones, M., R.C. McKinstry, A. Perry, J.R. Leonard, T.S. Park, and J.B. Rubin. 2008. Use of thalidomide to diminish growth velocity in a life-threatening congenital intracranial hemangioma. J. Neurosurg. Pediatr. 2:125–129. 10.3171/ped/2008/2/8/125 18671617 PMC2737696

[35] Shek, L.P., Y.S. Lee, B.W. Lee, and T.J. Lehman. 1999. Thalidomide responsiveness in an infant with Behçet’s syndrome. Pediatrics. 103:1295–1297. 10.1542/peds.103.6.1295 10353947

[36] Chen, T.L., G.B. Vogelsang, B.G. Petty, R.B. Brundrett, D.A. Noe, G.W. Santos, and O.M. Colvin. 1989. Plasma pharmacokinetics and urinary excretion of thalidomide after oral dosing in healthy male volunteers. Drug Metab. Dispos. 17:402–405. 10.1016/s0090-9556(25)07646-9 2571480

[37] Bai, N., X.Y. Cui, J. Wang, C.G. Sun, H.K. Mei, B.B. Liang, Y. Cai, X.-J. Song, J.-K. Gu, and R. Wang. 2013. Determination of thalidomide concentration in human plasma by liquid chromatography-tandem mass spectrometry. Exp. Ther. Med. 5:626–630. 10.3892/etm.2012.847 23404219 PMC3570145

[38] Murakami, H., K. Shimizu, M. Sawamura, K. Suzuki, I. Sugiura, H. Kosugi, C. Shimazaki, M. Taniwaki, M. Abe, and T. Takagi. 2009. Phase II and pharmacokinetic study of thalidomide in Japanese patients with relapsed/refractory multiple myeloma. Int. J. Hematol. 89:636–641. 10.1007/s12185-009-0314-5 19399582

[39] Hopewell, S., A.W. Chan, G.S. Collins, A. Hrobjartsson, D. Moher, K.F. Schulz, R. Tunn, R. Aggarwal, M. Berkwits, J.A. Berlin, N. Bhandari, . 2025. CONSORT 2025 statement: Updated guideline for reporting randomized trials. Nat. Med. 31:1776–1783. 10.1038/s41591-025-03635-5 40229553

[40] Simon, M., B. Pariente, J. Lambert, J. Cosnes, Y. Bouhnik, P. Marteau, M. Allez, J.-F. Colombel, and J.-M. Gornet. 2016. Long-term outcomes of thalidomide therapy for adults with refractory Crohn’s disease. Clin. Gastroenterol. Hepatol. 14:966–972.e2. 10.1016/j.cgh.2015.10.034 26598226

[41] FDA US . 2023. Rare Disease: Considerations for the Development of Drugs and Biological Products Guidance for Industry.

[42] EMA . 2006. Committee for Medicinal Products for Human Use. Guideline on Clinical Trials in Small Populations.

[43] Turner, D., A.R. Otley, D. Mack, J. Hyams, J. de Bruijne, K. Uusoue, T.D. Walters, M. Zachos, P. Mamula, D.E. Beaton, . 2007. Development, validation, and evaluation of a pediatric ulcerative colitis activity index: A prospective multicenter study. Gastroenterology. 133:423–432. 10.1053/j.gastro.2007.05.029 17681163

[44] Mohty, B., J. El-Cheikh, I. Yakoub-Agha, P. Moreau, J.L. Harousseau, and M. Mohty. 2010. Peripheral neuropathy and new treatments for multiple myeloma: Background and practical recommendations. Haematologica. 95:311–319. 10.3324/haematol.2009.012674 20139393 PMC2817035

[45] Kawai, T., K. Arai, S. Harayama, Y. Nakazawa, F. Goto, T. Maekawa, E. Tamura, T. Uchiyama, and M. Onodera. 2015. Severe and rapid progression in very early-onset chronic granulomatous disease-associated colitis. J. Clin. Immunol. 35:583–588. 10.1007/s10875-015-0180-2 26233238

[46] Pabla, B.S., and D.A. Schwartz. 2020. Assessing severity of disease in patients with ulcerative colitis. Gastroenterol. Clin. North Am. 49:671–688. 10.1016/j.gtc.2020.08.003 33121688 PMC7510557

[47] HHS US . 2009. Common Terminology Criteria for Adverse Events (CTCAE) Version 4.0. National Cancer Institute, National Institutes of Health, Bethesda, MD.

