# Supplementary material for: Thalidomide for CGD-related inflammatory bowel disease: A randomized, double-blind trial
Source: J Hum Immun. 2026 Jun 23;2(5):e20250256. doi: 10.70962/jhi.20250256 (PMC13289576; doi:10.70962/jhi.20250256)
Supplement: Table S1 — shows PUCAI and PGA for individual patients in the trial. [file jhi_20250256_tables1.doc]

Supplementary Table 1. PUCAI and PGA for individual patients in the trial

| Parameter | Phase | Time point | Thalidomide (n=3) | | |  | Placebo (n=5) | | | | |
| --- | --- | --- | --- | --- | --- | --- | --- | --- | --- | --- | --- |
|  |  |  | T1 | T2 | T3 |  | P1 | P2 | P3 | P4 | P5 |
| PUCAI | Blinded phase | Start | 15 | 45 | 25 |  | 50 | 35 | 40 | 35 | 25 |
|  |  | End * | 75 | 25 | 15 |  | 60 | 25 | 60 | 20 | 15 |
|  | Extension phase | Start | 75 | 25 | 15 |  | 60 | 25 | 60 | 20 | 15 |
|  |  | End * | 85 | 15 | 5 |  | 0 | 5 | 5 | 5 | 45 |
| PGA | Blinded phase | Start | Mild | Moderate | Mild |  | Moderate | Moderate | Moderate | Mild | Mild |
|  |  | End * | Severe | Mild | Mild |  | Moderate | Mild | Moderate | Mild | Mild |
|  | Extension phase | Start | Severe | Mild | Mild |  | Moderate | Mild | Moderate | Mild | Mild |
|  |  | End * | Severe | Mild | Inactive |  | Inactive | Inactive | Inactive | Inactive | Moderate |

* PUCAI and PGA were evaluated at the end of each phase or at discontinuation.

PUCAI, Pediatric Ulcerative Colitis Activity Index; PGA, Physician Global Assessment
