# Supplementary material for: Thalidomide for CGD-related inflammatory bowel disease: A randomized, double-blind trial
Source: J Hum Immun. 2026 Jun 23;2(5):e20250256. doi: 10.70962/jhi.20250256 (PMC13289576; doi:10.70962/jhi.20250256)
Supplement: Table S2 — shows schedule for the study visits and data collection. [file jhi_20250256_tables2.docx]

**Supplementary Table 2. Schedule for the study visits and data collection**

|  | Screening phase | Blinded phase  (12 weeks) | | | | | | |  | Extension phase  (12 weeks) | | | |  | Follow-up phase  (4 weeks) |
| --- | --- | --- | --- | --- | --- | --- | --- | --- | --- | --- | --- | --- | --- | --- | --- |
| Visit (Week) |  | 0 | 2 | 4 | 6 | 8 | | 12 |  | 14 | 16 | 20 | 24 |  | 28 |
| Obtain informed consent | X |  |  |  |  | X | | |  |  |  |  |  |  |  |
| Clinical assessments* | X | X | X | X | X | X | | X |  | X | X | X | X |  | X |
| Endoscopy | X |  |  |  |  |  | |  |  |  |  |  | X |  |  |
| Investigation drug administration |  | X | X | X | X | X | | X |  | X | X | X | X |  |  |
| Blood/urine tests† | X | X |  | X |  |  | | X |  |  | X |  | X |  | X |
| Vital signs‡ |  | X | X | X | X | X | | X |  | X | X | X | X |  | X |
| Stool occult blood tests§ | X | X | X | X | X | X | | X |  | X | X | X | X |  | X |
| PUCAI | X | X | X | X | X | X | | X |  | X | X | X | X |  | X |
| PGA | X | X | X | X | X | X | | X |  | X | X | X | X |  | X |
| ECG |  | X |  |  |  |  | | X |  |  |  |  | X |  |  |
| Nerve conduction tests |  | X |  |  |  | X | | |  |  |  |  | X |  | X |
| Adverse event assessment |  | X | X | X | X | X | X | |  | X | X | X | X |  | X |
| Pregnancy tests of participants¶ | X X | X |  | X |  | X | X | |  |  | X | X | X |  | X |
| Pregnancy tests of guardian# | X | X | X (at time of contact with investigational drug) | | | | | | | | | | | | |

* Clinical assessments include clinical history/past history/complications/treatments at screening, and clinical history/examination for all periods of the trial.

† Blood tests include *Aspergillus* antigen, *Candida* antigen, and beta-D-glucan.

‡ Vital signs include blood pressure, heart rate, and body temperature.

§ Occult blood tests were performed for the mild CGD-IBD patients who were occult blood-positive but did not have bloody stools.

¶ Pregnancy tests were conducted in all female patients of reproductive age.

# Pregnancy tests were conducted on all female guardians of reproductive age.

All investigators remained unaware of assignments until all data were finalized after study completion. However, investigators could request disclosure of a participant’s allocation code in urgent cases, such as pregnancy or a serious adverse event risking death.

PUCAI, Pediatric Ulcerative Colitis Activity Index; PGA, Physician Global Assessment; ECG, electrocardiogram.
